# The crucial roles of N^6^-methyladenosine (m^6^A) modification in the carcinogenesis and progression of colorectal cancer

**DOI:** 10.1186/s13578-021-00583-8

**Published:** 2021-04-09

**Authors:** Zhihao Fang, Yiqiu Hu, Jinhui Hu, Yanqin Huang, Shu Zheng, Cheng Guo

**Affiliations:** 1 Cancer Institute (Key Laboratory of Cancer Prevention and Intervention, China National Ministry of Education), The Second Affiliated Hospital, Zhejiang University School of Medicine, Hangzhou, 310009 Zhejiang China; 2 Department of Colorectal Surgery, Sir Run Run Shaw Hospital, Zhejiang University School of Medicine, Hangzhou, 310016 Zhejiang China

**Keywords:** N^6^-methyladenosine, RNA methylation, Coding and noncoding RNA, Colorectal cancer

## Abstract

As the predominant modification in RNA, N^6^-methyladenosine (m^6^A) has attracted increasing attention in the past few years since it plays vital roles in many biological processes. This chemical modification is dynamic, reversible and regulated by several methyltransferases, demethylases and proteins that recognize m^6^A modification. M^6^A modification exists in messenger RNA and affects their splicing, nuclear export, stability, decay, and translation, thereby modulating gene expression. Besides, the existence of m^6^A in noncoding RNAs (ncRNAs) could also directly or indirectly regulated gene expression. Colorectal cancer (CRC) is a common cancer around the world and of high mortality. Increasing evidence have shown that the changes of m^6^A level and the dysregulation of m^6^A regulatory proteins have been implicated in CRC carcinogenesis and progression. However, the underlying regulation laws of m^6^A modification to CRC remain elusive and better understanding of these mechanisms will benefit the diagnosis and therapy. In the present review, the latest studies about the dysregulation of m^6^A and its regulators in CRC have been summarized. We will focus on the crucial roles of m^6^A modification in the carcinogenesis and development of CRC. Moreover, we will also discuss the potential applications of m^6^A modification in CRC diagnosis and therapeutics.

## Background

Colorectal cancer (CRC) is the third most common malignant tumor in the world and it is of the second highest mortality [1]. Although the survival time of CRC patients has increased in recent years by improving basic diagnosis and treatment strategies, the mortality of CRC is still at a relatively high level. Due to the lack of reliable early diagnosis biomarkers and the asymptomatic disease progression, some of the diagnosed CRC patients are in advanced clinical stages, which results in death of approximately 900,000 patients every year [2]. Surgical resection is a possible cure strategy, but only for a few patients with advanced CRC, and postoperative tumor recurrence is very common. Due to the difficulties in early diagnosis and limited treatment options, CRC remains a challenging disease. It is generally believed that, genomic instability (e.g., chromosomal instability, DNA-repair defects and aberrant DNA/RNA methylation), mutational inactivation of tumor suppressor genes (e.g., APC, TP53 and TGF-β), and activation of oncogenes (e.g., RAS, BRAF and PI3K) are all involved in the occurrence and development of CRC [3], but the underlying molecular mechanisms remain largely unexplored. Thus, understanding the molecular mechanisms of CRC occurrence and progression more clearly is essential for advancing the diagnosis and treatment in the future.

Epigenetic modifications such as DNA methylation and histone modification have participated in many biological processes, and epigenetic dysregulation is involved in a variety of diseases including cancer. Recently, chemical modifications of RNA have drawn increasing attention since these modifications could fine-tune the structure and function of RNA, and this has leaded to the proposal of RNA epigenetics/epitranscriptomics [4]. As a typical form of RNA modification, N^6^-methyladenosine (m^6^A) was found both in coding RNA and noncoding RNAs (ncRNAs). The level of m^6^A modification is dynamic and reversible, and this is mainly mediated by the m^6^A methyltransferases and demethylases. The m^6^A modification is recognized by different reader proteins and exerts its unique biological function. Specifically, through targeted regulation of RNA fate, deregulation of m^6^A modulators is related to immunomodulation [5], cancer stemness [6], epithelial-mesenchymal transition (EMT) [7] and cancer metabolism [8]. Multiple m^6^A regulatory proteins are recently reported to be abnormally regulated and act either as oncogenes or tumor suppressors in CRC. In this review, we summarized the latest findings about the impacts of m^6^A modification located on different types of RNA on CRC initiation and progression. Moreover, we discussed the potential implications of m^6^A modification in CRC diagnosis and therapeutics.

## N^6^-methyladenosine

N^6^-methyladenosine (m^6^A) modification refers to the addition of a methyl group at the N^6^ position of adenosine. This is an evolutionarily conservative RNA modification that can be found in most organisms, from bacteria to mammals [9]. M^6^A modification is considered to be the most common chemical modification in eukaryotic mRNA and lncRNA [10], and 0.1–0.4% of all adenosines in total cellular RNAs with an average of 3–5 m^6^A modifications per mRNA [11]. The action-sites of m^6^A modification are not random, and they have a typical consensus sequence RRACH (R = G, A or U; H = A, C or U). Moreover, m^6^A modification highly occurs in 3′ untranslated region (3′-UTR), near the stop codon region, and long internal exons [10, 12].

### ***Reversible m***^***6***^***A modification***

Similar to DNA, RNA can also be methylated and demethylated by specific methyltransferases and demethylases, respectively. RNA m^6^A modification was previously considered to be static, discrete, and used to fine-tune RNA structure and function. However, with the discovery of METTL3/METTL14/WTAP methyltransferase complex [13] and demethylase FTO [14] and ALKBH5 [15], investigations on m^6^A modification have returned to the forefront because these findings indicate that m^6^A modification is reversible and can be dynamically adjusted, implying the potential of these regulatory proteins in regulating biological processes.

### ***Writers, erasers and readers of m***^***6***^***A***

**Fig. 1:**
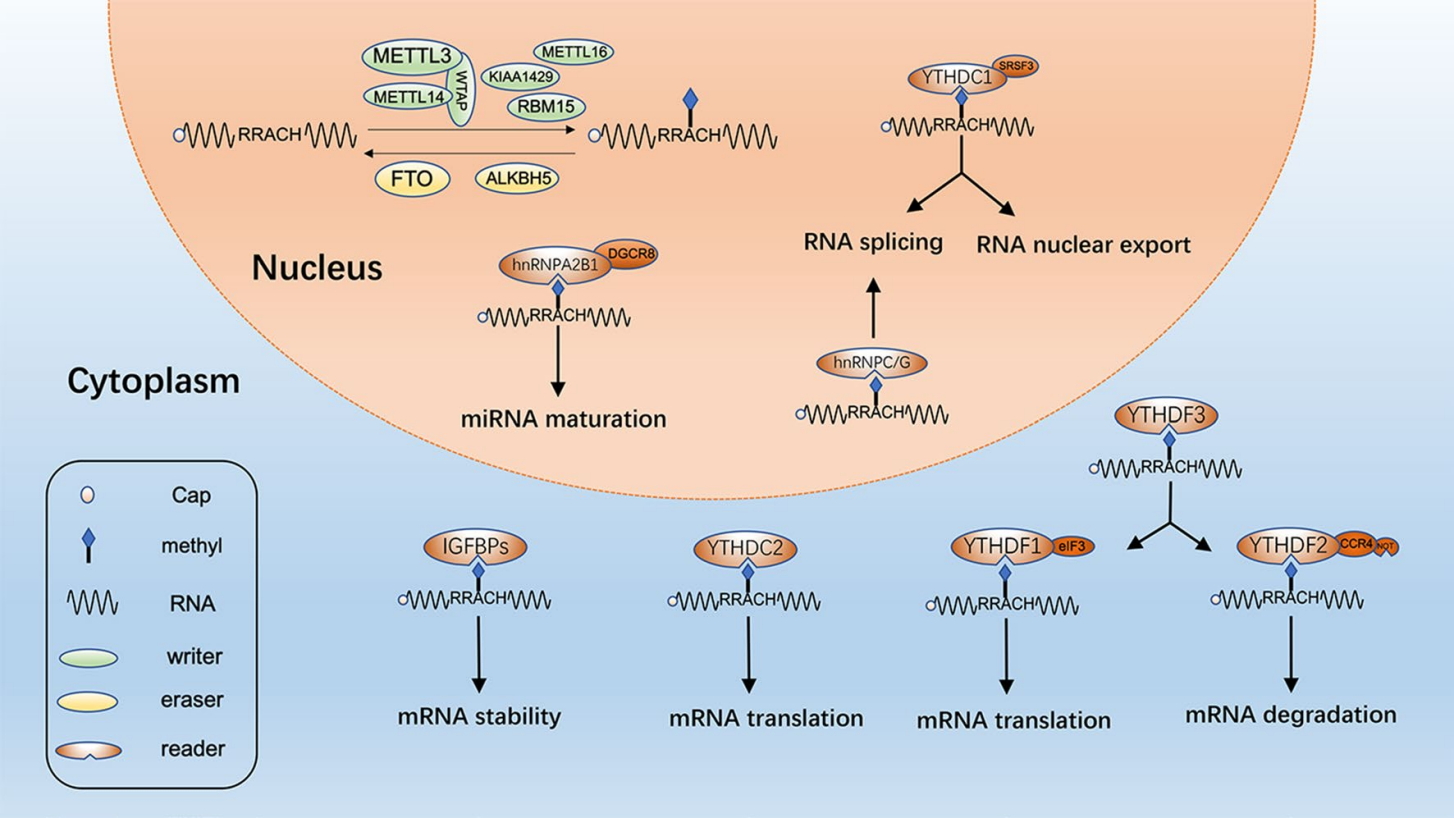
The mechanism of m^6^A regulation. The level of m^6^A modification is mediated by the m^6^A writer and eraser proteins, and the m^6^A modification is recognized by different reader proteins to influence RNA fate

The biological effects of m^6^A are mainly mediated by the proteins of writer, eraser and reader (Fig. 1). As mentioned above, m^6^A modification is dynamic and reversible, and this indicates RNA can be methylated by methyltransferases (writers) and demethylated by demethylases (erasers). Writers mainly consist of METTL3 and METTL14 and their cofactors WTAP. Both of METTL3 and METTL14 contain an *S*-adenosylmethionine-binding (SAM-binding) motif to play the role of transferring methyl group to adenosine at N^6^ position [16]. METTL3 is the major catalytic enzyme and the main function of METTL14 is to stabilize METTL3 and recognize target RNA [17, 18]. WTAP can regulate m^6^A methylation complex which helps METTL3 and METTL14 to be located in nuclear spots [19]. Besides, METTL16 [20], KIAA1429 [21] and RBM15 [22] were also identified as m^6^A writers.

Erasers, which can achieve the function of demethylation, mainly include FTO and ALKBH5. FTO was identified as the first m^6^A demethylase, and this major breakthrough leads to great prosperity in the realm of RNA epigenetics. FTO can sequentially oxidize m^6^A to N^6^-hydroxymethyladenosine and N^6^-formyladenosine, and then N^6^-formyladenosine can be hydrolyzed to adenosine easily [14]. ALKBH5, an FTO homologue, can directly oxidize m^6^A to adenosine [15].

Apart from writers and erasers, another group is readers, which can recognize m^6^A modification and exert different biological functions. One type of readers with the YT521B homology (YTH) domain contains YTHDF1, YTHDF2, YTHDF3, YTHDC1 and YTHDC2. They all have specific m^6^A binding domains and preferentially bind to m^6^A modification sites in RNA with RRm^6^ACH sequence [23]. Among these, YTHDF2 was firstly identified and it can influence mRNA stability. YTHDF2 accelerates the degradation of mRNA by sending m^6^A-mediated mRNAs to mRNA decay sites (such as P-bodies) [24], and it can also recruit the CCR4-NOT deadenylase complex to promote mRNA degradation [25]. YTHDF1 has shown the ability to promote mRNA translation efficiency by interacting with translation initiation factor eIF3 [26]. YTHDF3 can be fine-tuned to balance the different effects of YTHDF1 and YTHDF2 on mRNA [27, 28]. YTHDC1 can mediate RNA splicing and control the nuclear export of its targets by interacting with SRSF3 [29]. YTHDC2 can interact with RNA helicase to promote the extension of the translation process [30]. Moreover, readers also contain IGF2BP1/2/3 and three heterogeneous nuclear ribonucleoproteins (hnRNPs), including hnRNPC, hnRNPG and hnRNPA2B1. IGF2BP proteins specifically recognize m^6^A-containing transcripts and stabilize them. For hnRNPs, it has been reported that hnRNPC/G can influence mRNA localization and alternative splicing [31], and hnRNPA2B1 can promote microRNA maturation with the help of DGCR8, which is an RNA-binding protein that recognizes pri-miRNA hairpin [32].

## Function of m^6^A on coding and noncoding RNAs

### ***Function of m***^***6***^***A on mRNA***

**Fig. 2:**
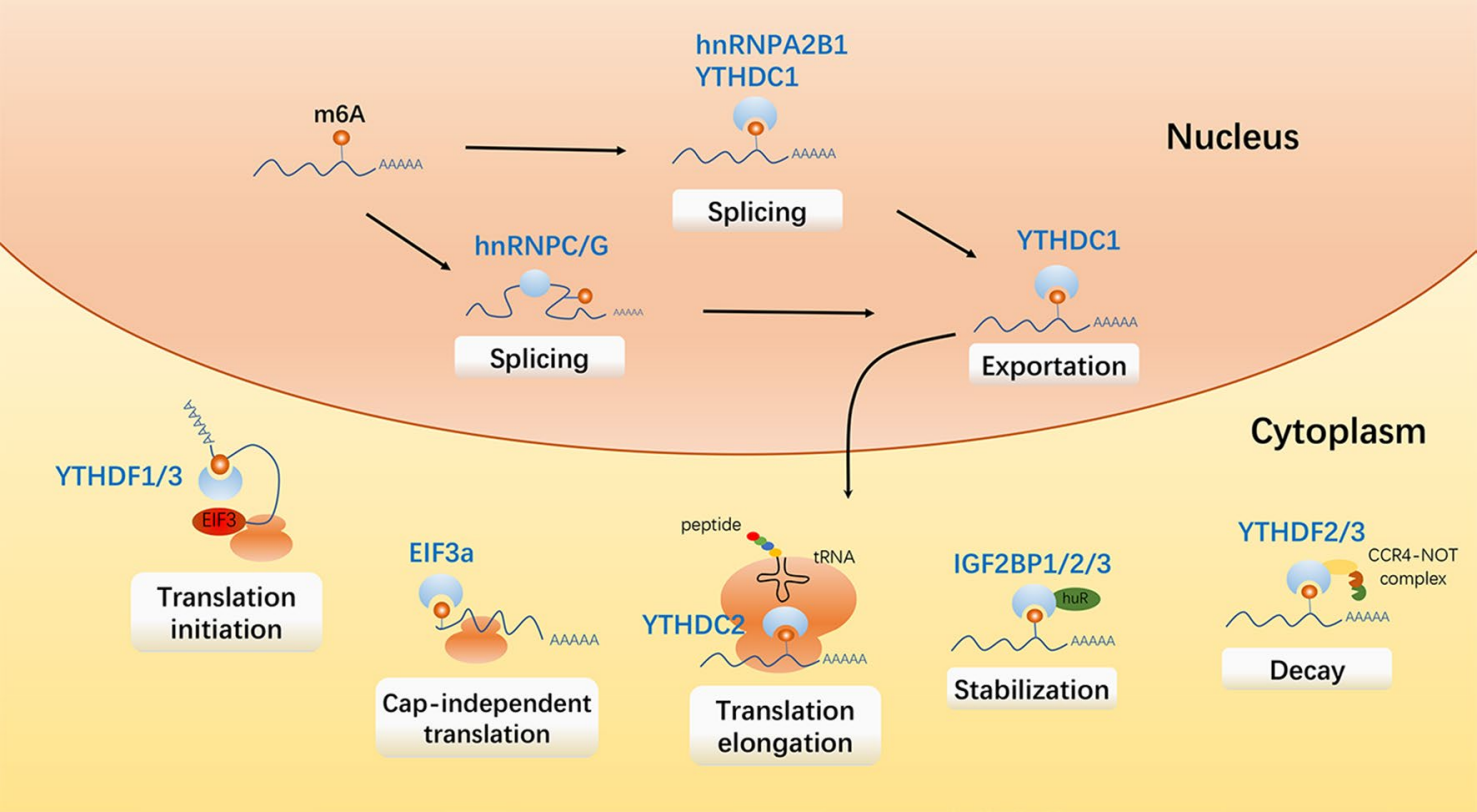
Functions of m^6^A modification on mRNA. M^6^A modification undergo alternative splicing with the recruitment of splicing factors to m^6^A sites or flanking sequences. After splicing, m^6^A-containing mRNAs are recognized by YTHDC1 and exported into cytoplasm. M^6^A on mature mRNAs affects mRNA stability, translation initiation, and translation elongation

Gene expression is strictly controlled at four levels, i.e., transcription, post-transcription, translation and post-translation. M^6^A modification deposits on natural RNA transcripts during transcription and affects gene expression after transcription by altering RNA structure or through specific recognition by m^6^A binding proteins [33] (Fig. 2).

The m^6^A writers and erasers are located in nuclear speckles, where they are related to mRNA splicing factors, suggesting that m^6^A is functionally related to mRNA splicing [14, 15, 34]. Specifically, m^6^A on the pre-mRNA can exert mRNA splicing function by recruiting hnRNPB2A1 or changing the local structure to increase the accessibility to the splicing factor hnRNPC/hnRNPG [35, 36]. In addition, m^6^A can also recruit the splicing factor SRSF3 by binding to YTHDC1 to complete the splicing of exons [37]. YTHDC1 can also affect the exportation of m^6^A modified mRNA from nucleus to cytoplasm and thus affects its transcription process [29].

Mature mRNA in the cytoplasm can be loaded onto ribosomes for active translation, or sorted onto messenger ribonucleoprotein (mRNP) foci for degradation or storage. The YTH domain-containing family proteins (YTHDFs) tend to accelerate metabolism of m^6^A-modified mRNAs. YTHDF1 selectively recognizes m^6^A and interacts with initiation factor eIF3 to promote translation initiation and protein synthesis [26]. On the contrary, YTHDF2 brings m^6^A modified translatable mRNA to the site of mRNA decay and recruits CCR4-NOT complex to trigger mRNA degradation [25, 38]. YTHDF3 cooperates with YTHDF1 to promote mRNA translation and promotes the degradation of m^6^A-containing mRNA by interacting with YTHDF2 [27, 28]. In contrast to the promoting degradation function of YTHDF2, proteins of the IGF2BP family interact with ELAVL1 (also known as HuR), MATR3, and PABPC1 to protect m^6^A-modified mRNA in P-body and stress granules from degradation and promote their translation [39]. Since m^6^A is usually enriched near the stop codon, the role of m^6^A in promoting translation initiation is achieved through a closed-loop model, in which the cyclization of mRNA is accomplished via the interaction between the elFs and METTL3 or YTHDF1 bound to the m^6^A site near the stop codon [26, 40]. It is also reported that the m^6^A modification in 5′UTR can promote cap-independent translation by recruiting eIF3a to a nearby translation start site [41]. In the translation extension stage, the m^6^A modification on mRNA can hinder tRNA accommodation, thereby disturbs the translation extension dynamics [42]. Besides, m^6^A modification on mRNA can bind to YTHDC2 to positively regulate translation elongation [30].

### ***Function of m***^***6***^***A on ncRNAs***

**Fig. 3:**
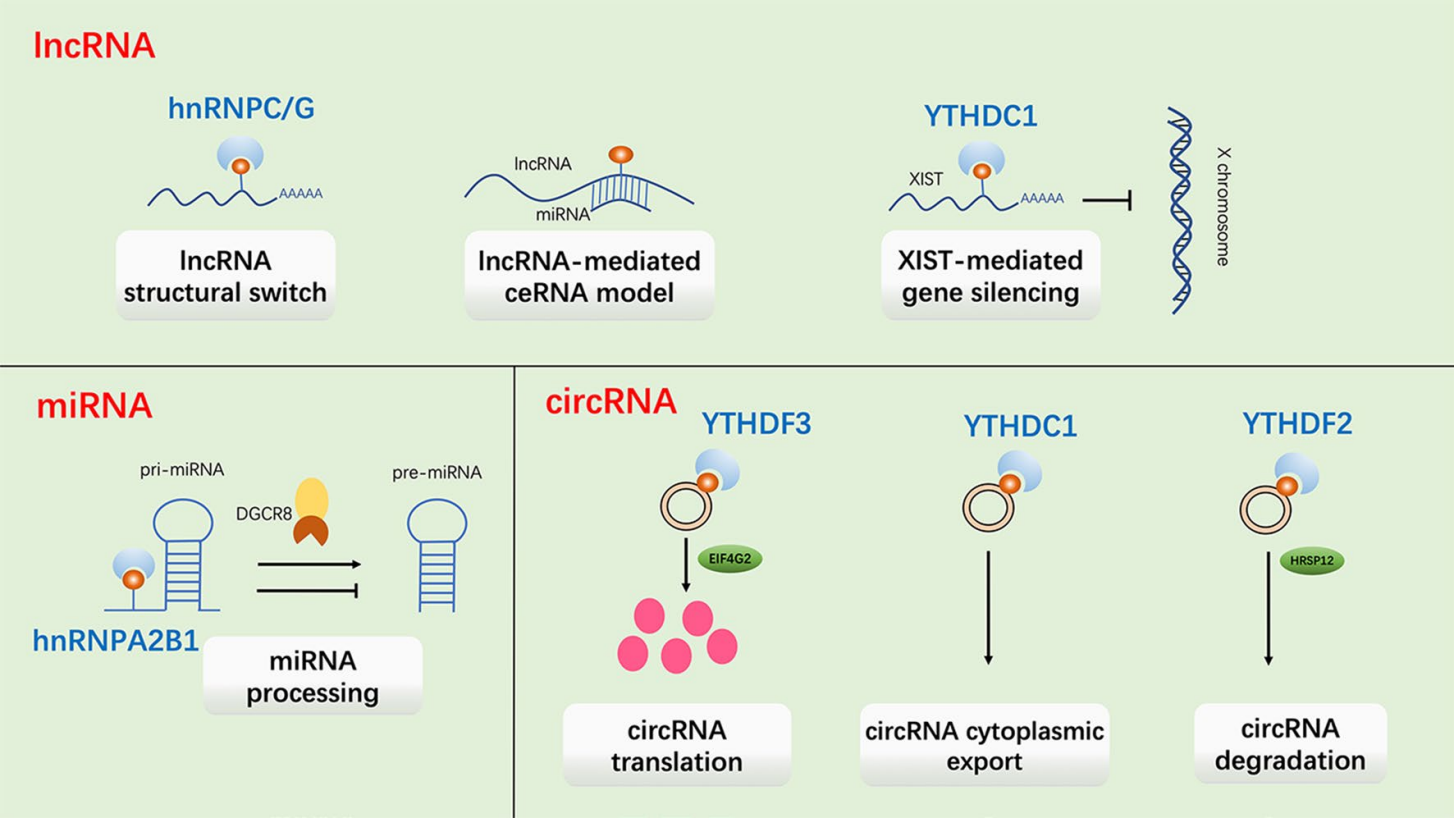
Functions of m^6^A modification on non-coding RNA. M^6^A on lncRNA plays roles in RNA structural switch, lncRNA-mediated ceRNA model and XIST-mediated gene silencing. Presence of m^6^A on pri-miRNA facilitates or inhibits miRNA processing. In circRNA, m^6^A could promote circRNA translation, circRNA cytoplasmic export and circRNA degradation

Although ncRNAs cannot be directly translated into protein, they have special functions in regulating gene expression, and can be divided into long non-coding RNA (lncRNA) and small non-coding RNA according to whether the length exceeds 200 nt. Previous studies indicated that m^6^A modification also played important roles in the expression and functions of ncRNAs, including lncRNA, miRNA and circRNA [43–46] (Fig. 3).

The m^6^A methylation on lncRNA was usually located on the poly (A) region. Due to the lack of coding ability, lncRNA regulates gene expression by acting as an RNA structural switch, participating in the lncRNA-mediated competitive endogenous RNA (ceRNA) model or promoting X-inactive specific transcript (XIST) mediated gene silencing. lncRNA-mediated ceRNA model means that lncRNA acts as a competitive RNA of miRNA to affect its function. It was reported that m^6^A modification acted as a structural switch to affect RNA–protein interactions by modulating the structure of several RNAs, including metastasis-associated lung adenocarcinoma transcript (MALAT1) [47], which is a lncRNA and has been consistently associated with the progression of various cancers. Several studies have proposed lncRNA-mediated ceRNA model, in which lncRNA LINC01234 and SNHG6-003 act as a competitive endogenous RNA to regulate the activity and biological function of miRNAs [48, 49]. In addition, WTAP and RBM15B have been identified as XIST-associated protein and both of them are necessary for XIST-mediated transcriptional repression [22, 50].

MiRNA is a highly abundant small noncoding RNA and its functions mainly include gene silencing and post-transcriptional gene expression regulation. The primary transcript of miRNA (pri-miRNA) transcribed from DNA undergoes a series of cleavage processes to generate hairpin precursors, precursor miRNA (pre-miRNA) and mature miRNA successively. The pri-miRNAs are rich in canonical m^6^A motif (GGAC), and most of them can be methylated by METTL3 [51]. The pri-miRNAs labeled with m^6^A can be recognized by hnRNPA2B1, which can interact with DGCR8 and promote miRNA processing [32]. Interestingly, no changes were observed in the primary transcription products. After FTO knockdown, several mature miRNAs were down-regulated [52], indicating that m^6^A has a negative impact on the stability of miRNAs. Therefore, the amount of mature miRNA can be altered by knocking down or overexpressing m^6^A regulatory proteins to affect the level of m^6^A.

CircRNA is a new type of ncRNA, which can form a covalently closed continuous loop structure by back splicing. Recently, it has been found that m^6^A modification is also prevalent in circRNA and shares the readers and writers with that in mRNA [53]. Alike in mRNA, recognition of m^6^A-modified circRNA by YTHDF2 can also promote circRNA degradation. Park et al. reported that a subset of m^6^A-modified circRNAs combined with YTHDF2 was selectively downregulated by RNase P/MRP [54]. Besides, m^6^A promotes cytoplasmic export of circRNAs, Chen et al. found that YTHDC1 could export circNSUN2 from the nucleus to the cytoplasm to stabilize HMGA2 mRNA [55]. M^6^A can also drive circRNA-related protein synthesis by recruiting YTHDF3 and initiation factor eIF4G2 [56].

Interestingly, several studies reported that ncRNAs can affect m^6^A regulators reversely. Wang et al. [57] found that lncRNA LINRIS (Long Intergenic Noncoding RNA for IGF2BP2 Stability) blocks K139 ubiquitination of IGF2BP2 to maintain its stability so as to promote the aerobic glycolysis in CRC. As for miRNA, it was reported that FTO is negatively regulated by miR-1266 and lowly expressed miR-1266 promotes the occurrence and progression of CRC by directly targeting FTO [58]. Moreover, Yue et al. [59] found that miR-96 could enhance the expression of FTO indirectly by down-regulating AMPKα2 to promote occurrence and progression of CRC.

## The implications of m^6^A modification in CRC

**Fig. 4:**
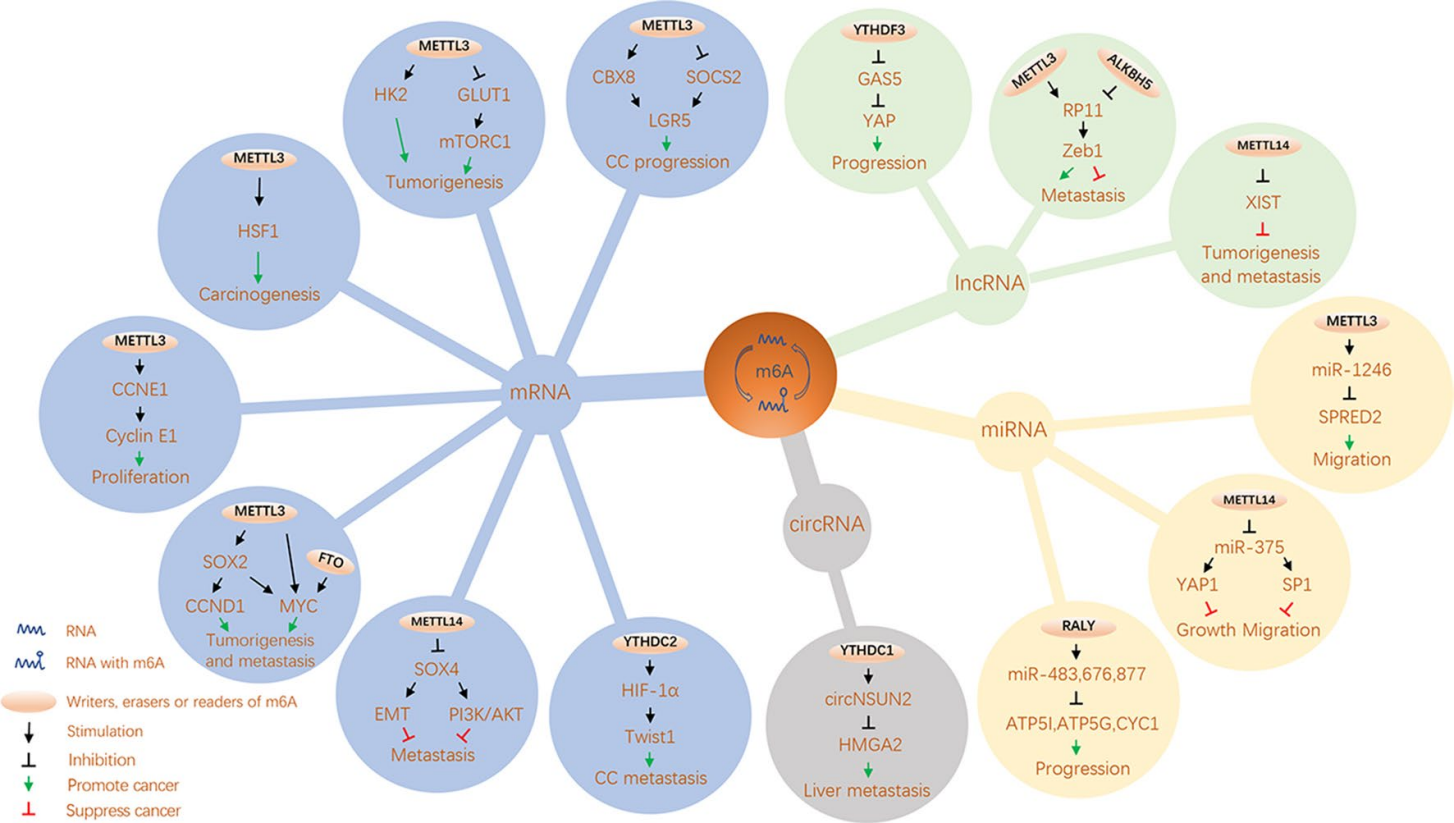
The molecular mechanism of m^6^A on different types of RNA involved in CRC. METTL3, METTL14, FTO and YTHDC2 regulate m^6^A modification on mRNA to affect CRC initiation and progression. METTL3, METTL14, ALKBH5 and YTHDF3 regulate m^6^A modification on lncRNA to affect CRC. METTL3, METTL14 and hnRNPCL2 regulate m^6^A modification on miRNA to affect CRC. YTHDC1 regulates m^6^A modification on circRNA to affect CRC

In recent years, continuous efforts have been devoted to revealing the important roles of the imbalance of m^6^A modification and m^6^A regulatory protein in CRC. Considering the key role of m^6^A regulatory protein in mediating the occurrence and development of CRC, we reviewed the tumor-promoting and anti-tumor effects of several important m^6^A regulatory proteins on CRC (Table 1). Interestingly, we also found that m^6^A modifications could play similar roles by targeting different types of RNA molecules. And we summarized these mechanisms in Fig. 4.

**Table 1:** The roles of different m6A regulators in CRC

m^6^A regulators	Genes	Location	Role	Mechanism	Function	Refs
Writers						
METTL3	SOX2	mRNA	Oncogene	Stabilize SOX2 mRNA	Promoting CRC tumorigenesis and metastasis	[60]
	MYC	mRNA	Oncogene	Enhance expression of MYC	Promoting CC cells proliferation	[61]
	CCNE1	mRNA	Oncogene	Stabilize CCNE1 mRNA	Promoting CRC cells proliferation	[62]
	HK2/GLUT1	mRNA	Oncogene	Stabilize HK2 and GLUT1 mRNA	Promoting CRC tumorigenesis	[63]
	GLUT1	mRNA	Oncogene	Promote GLUT1 mRNA translation	Promoting CRC development	[64]
	CBX8	mRNA	Oncogene	Stabilize CBX8 mRNA	Promoting CC stemness and chemoresistance	[65]
	SOCS2	mRNA	Oncogene	Degrade SOCS2 mRNA	Promoting CC cells proliferation	[66]
	HSF1	mRNA	Oncogene	Promote HSF1 mRNA translation	Promoting CRC development	[67]
	RP11	lncRNA	Oncogene	Promote the nuclear accumulation of RP11	Promoting CRC cells migration	[68]
	miR-1246	miRNA	Oncogene	Promote miR-1246 maturation	Promoting CRC cells migration and invasion	[69]
	–	–	Tumor suppressor	Decrease the activation of p-p38 and p-ERK	Inhibiting CRC proliferation and migration	[70]
METTL14	SOX4	mRNA	Tumor suppressor	Degrade SOX4 mRNA	Inhibiting CRC metastasis	[71]
	XIST	lncRNA	Tumor suppressor	Mediate the degradation of XIST	Inhibiting CRC tumorigenicity and metastasis	[72]
	miR-375	miRNA	Tumor suppressor	Reduce miR-375 maturation	Inhibiting CRC cell growth, migration	[73]
WTAP	–	–	Oncogene	Promote Wnt signaling pathway	Promoting CC development	[77]
Erasers						
FTO	MYC	mRNA	Oncogene	Enhance expression of MYC	Promoting CRC occurrence and progression	[59]
ALKBH5	RP11	lncRNA	Tumor suppressor	Decrease the nuclear accumulation of RP11	Inhibiting CRC cells migration	[68]
Readers						
YTHDF1	–	–	Oncogene	Promote Wnt/β-catenin pathway	Promoting CRC cells tumorigenicity	[74]
YTHDF3	GAS5	lncRNA	Oncogene	Facilitate the degradation of GAS5	Promoting CRC progression	[75]
YTHDC1	circNSUN2	circRNA	Oncogene	Facilitate circNSUN2 nuclear export	Promoting CRC cells liver metastasis	[55]
YTHDC2	HIF-1α	mRNA	Oncogene	Stabilize HIF-1α mRNA	Promoting CC cells proliferation	[76]
hnRNPCL2	miR-483,676,877	miRNA	Oncogene	Promote the miRNAs procession	Promoting CRC progression	[78]

### METTL3 promotes CRC occurrence and progression

METTL3, as the most common m^6^A methyltransferase, has been found to play oncogenic function in a variety of cancers, including CRC. Li et al. [60] found that METTL3, as an oncogene, can extend the half-life of sex determining region Y-box 2 (SOX2) mRNA relied on IGF2BP2 to maintain the expression of SOX2 and SOX2 target genes (CCND1, MYC and so on), which further promotes CRC progression. Similarly, another study suggested that METTL3 promotes CRC progression through enhancing c-myc proto-oncogene (MYC) expression in an m^6^A-IGF2BP1-dependent manner [61]. METTL3 could methylate 3′UTR of CCNE1 mRNA to stabilize it and make it translated to form more cyclin E1 protein, which promotes CRC cell proliferation [62]. Additionally, METTL3 was reported to be directly interacted with the 5′/3′UTR regions of Hexokinase 2 (HK2) mRNA relied on IGF2BP2/3, the 3′UTR region of Glucose transporter 1 (GLUT1) mRNA relied on IGF2BP2, stabilized them and then further activated the glycolysis pathway to induce CRC tumorigenesis [63]. As mentioned above, METTL3-mediated different m6A-modified mRNA molecules were recognized by IGF2BP1/2/3, and ultimately played the same role in promoting the translation of mRNA molecules, although they used different mechanisms to protect the stability of mRNA molecules. Further, a recent study reported that METTL3 directly induced m^6^A-GLUT1-mTORC1 axis to promote CRC development [64]. Zhang et al. [65] found that METTL3 relied on IGF2BP1 to stabilize chromo box 8 (CBX8) mRNA which can inhibit the chemosensitivity and maintain the stemness of colon cancer (CC) by promoting the expression of leucine rich repeat containing G protein-coupled receptor 5 (LGR5). Similarly, Xu and colleagues [66] found that the up-regulation of METTL3 leads to a decrease in the expression of suppressor of cytokine signaling 2 (SOCS2), and further promotes CC cell proliferation by inducing LGR5. Besides, METTL3 that favored by β-catenin could bind and activate Heat shock transcription factor1 (HSF1) mRNA translation initiation to promote CRC development [67].

Except METTL3 targeting downstream mRNA has been reported, METTL3 could also affect the progress of CRC by regulating the m6A modifications targeted ncRNAs. Wu et al. [68] found that RP11 as lncRNA was highly expressed in CRC tissues and was positively correlated with the tumor stage of patients. METTL3 can induce the formation of m^6^A modification in RP11 to increase the nuclear accumulation of RP11 which plays a role in preventing Zeb1 degradation. Zeb1 is an EMT-related transcription factor, and upregulation of Zeb1 is essential for RP11-induced cell invasion and metastasis. In addition, Peng and co-workers [69] reported that upregulated METTL3 could promote the maturation of pri-miR-1246 relied on DGCR8. Mature miR-1246 could decrease the expression of anti-oncogene SPRED2 to exert the effect of promoting CRC cells migration by interacting with the Raf/MEK/ERK pathway.

Overwhelming majority of studies believed that MELLT3 targeting mRNA and ncRNAs to play a role in promoting cancer in the occurrence and development of CRC. However, Deng et al. proposed that METTL3 acted as a tumor suppressor in the proliferation, migration and invasion of CRC cells through the p38/ERK pathway [70].

### METTL14 suppresses CRC occurrence and progression

Unlike METTL3, METTL14 has an opposite effect on the occurrence and development of CRC relied on the function of m^6^A reader YTHDF2. METTL14-mediated m^6^A modified RNA molecules are selectively recognized and bound by YTHDF2, thereby promoting the degradation of these RNA molecules. A recent study reported by Wang group shows that METTL14 promotes SOX4 mRNA degradation by relying on YTHDF2-dependent pathway, and this can inhibit CRC progression partly through SOX4-mediated EMT process and PI3K/Akt signals [71]. Similarly, Yang and colleagues [72] reported that METTL14 down-regulated the expression of XIST lncRNA through the m^6^A-YTHDF2-dependent pathway to exert tumor suppressive effect. Consistently, XIST expression was negatively correlated with the content of METTL14 and YTHDF2 in CRC tissue, and the reduction of METTL14 was related to the poor prognosis of CRC patients in clinical. Besides, Chen et al. [73] found the expression of METTL14 in CRC tissue was down-regulated and closely related to the overall survival of CRC patients. METTL14 knockdown would weaken the binding of DGCR8 to the primary miR-375 and thus reduce the expression level of miR-375. Specifically, they verified that METTL14 suppressed CRC cell growth via the miR-375/Yes-associated protein 1 (YAP1) pathway, and inhibited CRC cell migration and invasion through the miR-375/SP1 pathway.

### YTH family proteins promote CRC occurrence and progression

A study reported that knocking down the expression of YTHDF1 can significantly inhibit the tumorigenicity of CRC cells in vitro and the growth of mouse xenografts in vivo [74]. Moreover, Ni et al. [75] found that YTHDF3 was not only a new target of YAP, but also could promote the degradation of m^6^A modified lncRNA GAS5. GAS5 can promote the nuclear export of endogenous YAP and further promote its phosphorylation and subsequent ubiquitin-mediated YAP degradation, and thus the expression of lncRNA GAS5 in tissue of CRC patients is negatively correlated with the level of YAP protein. In summary, this study revealed the negative functional loop of the GAS5-YAP-YTHDF3 axis to explain the role of m^6^A modified GAS5 in the progression of CRC. In addition, Chen et al. [55] found that m^6^A modified circNSUN2 was often upregulated in tumor tissues and serum samples from CRC patients with liver metastasis, and could be used to predict poor survival of CRC patients. Besides, they clarified that m^6^A modification of circNSUN2 regulated cytoplasmic output through YTHDC1-dependent manner. It is worth noting that by forming the circNSUN2/IGF2BP2/HMGA2 RNA–protein ternary complex, increased cytoplasmic expression of circNSUN2 can enhance the stability of HMGA2 mRNA, which further leads to liver metastasis of CRC. Tanabe and colleagues [76] revealed that YTHDC2 can facilitate transcription factor hypoxia-inducible factor-1α (HIF-1α) protein express, thereby playing an important role in the metastasis of CRC by promoting epithelial-mesenchymal transition (EMT).

### ***Other m***^***6***^***A regulatory proteins affect CRC occurrence and progression***

Zhang et al. [77] reported that WTAP could serve as a novel oncogene in CRC by forming WTAP-WT1-TBL1 axis to mediate Wnt signaling pathway. Besides, Yue et al. [59] found that FTO could upregulate MYC via blocking its m^6^A modification, further promote CRC occurrence and progression. ALKBH5 was found to have the opposite function to METTL3, it could serve as a tumor suppressor gene in CRC by reducing the nuclear accumulation of RP11 and thus inhibiting the function of EMT [35]. In addition, Sun et al. [78] reported a novel RNA-binding protein, RALY (also known as hnRNPCL2) could mediate mitochondrial metabolism to promote CRC development. RALY could bind to m^6^A-modified pri-miRNAs to promote the post-transcriptional processing of a specific subset of miRNAs (miR-483, miR-676 and miR-877), which downregulate the expression of the metabolism-associated genes (ATP5l, ATP5G1, ATP5G3 and CYC1) to reprogramme mitochondrial metabolism in the cancer cell.

## Discussion

### ***The diagnosis potential of m***^***6***^***A for CRC***

Deregulation of m^6^A writer, eraser and reader proteins has been recently reported to be associated with increased probability of CRC occurrence and increased aggressiveness. Liu et al. [79] found that most genes related to m^6^A were significantly up-regulated in tumor tissues from CRC patients compared with normal tissues, but METTL14, YTHDF3 and ALKBH5 were down-regulated. Survival analysis showed that the expression levels of METTL14, METTL16 and FTO were positively related to the clinical prognosis of CRC patients but the expression levels of METTL3 and ALKBH5 were negatively related to it. In addition, Ji et al. [80] reported that the dysregulation of WTAP and FTO are significantly related to the progression of CRC, and YTHDC2 and ALKBH5 can predict the prognosis of CRC patients independently. Taken together, the expression of m^6^A and its regulators (writers, erasers, readers) may be a potential biomarker for molecular typing and prognosis prediction of CRC patients. A recent study demonstrated that m^6^A could be detected in the circulating tumor cells (CTCs) from lung cancer patients by liquid chromatography-tandem mass spectrometry, and the level of m^6^A was higher than that in whole blood [81]. This study indicates that analysis of m^6^A levels in CTC may become a new non-invasive cancer diagnostic method. Further studies to confirm whether the differences of m^6^A and m^6^A regulators can be observed in precancerous lesions are desirable, which will be helpful to evaluate the potential of these molecules as indictors for early detection of CRC.

### ***The therapeutic potential of m***^***6***^***A for CRC***

Previous studies have shown that dysregulation of m^6^A regulatory factors may be related to the development of chemotherapy resistance. For instance, Nishizawa et al. [82] reported that c-Myc promoted YTHDF1 expression to increase chemotherapy sensitivity for CRC. It was also reported that METTL3 participated in the upregulation of CBX8 which could promote stemness and suppress chemosensitivity through LGR5 [65]. These studies highlighted the important therapeutic value of targeted m^6^A modulators in drug-resistant tumors. In addition, targeting m^6^A and m^6^A regulators could also be a potential therapeutic strategy for radiotherapy and immunotherapy. A recent study found that the inhibition of METTL3/14 methylation modifications to STAT1 and IRF1 can enhance the sensitivity of anti-PD-1 therapy in pMMR/MSI-L CRC [83]. So far, no studies have explained the relationship between m^6^A modification and CRC radiotherapy sensitivity. Chemotherapy is commonly used in CRC and radiotherapy is an indispensable treatment for patients with low- and middle-level rectal cancer. PD-1/PD-L1-related immunotherapy has proven to be effective in many cancers including CRC [84]. It is important to identify the different therapeutic sensitivity of each patient via certain indicators, and m^6^A regulators could be such indicators. Therefore, more novel researches on m^6^A regulators and CRC treatment responses are urgently needed.

With further understanding of m^6^A modulators, it is more and more necessary to develop effective and specific m^6^A modulators inhibitors [85]. Although Rhein was found not to be an FTO-specific inhibitor, Huang et al. [86] revealed that meclofenamic acid (MA) showed high selectivity in inhibiting FTO over ALKBH5. Besides, it was reported that the proliferation and progression of acute myeloid leukemia in vivo was significantly reduced by treatment with FB23-2, a derivative of MA [87]. In another study, Shen et al. [63] found that 3-deazaadenosine (DAA), a chemical inhibitor of m^6^A, could more effectively inhibit the proliferation of CRC cells with high expression of METTL3. Sincerely, further molecular structure studies and large-scale chemical screening experiments are needed to develop specific inhibitors against dysregulated m^6^A regulatory proteins. Novel specific m^6^A regulatory protein inhibitors will not only enhance our understanding of the function of m^6^A modulators acted on carcinogenesis and progression of CRC, but also provide new and more favorable treatment strategies for CRC.

## Conclusion

RNA m^6^A modification plays vital roles in post-transcriptional regulation of gene expression, and it is involved in the occurrence and development of CRC. Dysregulation of m^6^A regulators can regulate the expression of downstream targets by mediating different RNA fate. In this review, we summarized recent studies on elucidation of the important roles of m^6^A modification played in the occurrence and development of CRC. Further investigations are desirable to elucidate the heterogeneity and complexity of m^6^A modification and m^6^A modulators in CRC carcinogenesis and progression. With the rapid development of m^6^A localization methods and m^6^A editing tools, the studies of m^6^A at the level of single nucleotide will be greatly promoted, and this will help researchers to better understand the roles of m^6^A in CRC tumorigenesis and development. Additionally, more efforts are required to identify new specific m^6^A modification for early diagnosis of CRC, and develop specific inhibitors of m^6^A modulators for better therapeutic purposes in future.

## Data Availability

Not applicable.

## Abbreviations

ALKBH5: Alpha-ketoglutarate-dependent dioxygenase alkB homolog 5
CBX8: Chromo box 8
ceRNA: Competitive endogenous RNA
CRC: Colorectal cancer
circRNA: Circular RNA
DGCR8: DiGeorge syndrome crisis area gene 8
EMT: Epithelial–mesenchymal transition
FTO: Fat mass and obesity-associated protein
GLUT1: Glucose transporter 1
HIF-1α: Hypoxia-inducible factor-1α
HK2: Hexokinase 2
hnRNP: Heterogeneous nuclear ribonucleoproteins
HSF1: Heat shock transcription factor1
IGF2BP1–3: Insulin like growth factor 2 binding protein 1–3
lncRNA: Long noncoding RNA
METTL14: Methyltransferase like 14
METTL3: Methyltransferase like 3
miRNA: MicroRNA
MYC: C-myc proto-oncogene
m^6^A: N6-methyladenosine
SOCS2: Suppressor of cytokine signaling 2
SOX2: Sex determining region Y-box 2
SOX4: Sex determining region Y-box 4
WTAP: WT1 associated protein
XIST: X-inactive specific transcript
YTH: YT521-B homology
YTHDC1–2: YTH domain containing protein 1–2
YTHDF1–3: YTH domain containing protein family
YAP: Yes-associated protein
